# Impact of early enteral nutrition on the prognosis of mechanically ventilated patients with chronic obstructive pulmonary disease: a retrospective cohort study based on the MIMIC-IV Database

**DOI:** 10.3389/fnut.2025.1620011

**Published:** 2025-08-13

**Authors:** Lamei Ouyang, Canmin Wang, Yunfeng Song

**Affiliations:** Department of Critical Care Medicine, The Affiliated Guangdong Second Provincial General Hospital of Jinan University, Guangzhou, Guangdong, China

**Keywords:** Early enteral nutrition, chronic obstructive pulmonary disease, prognosis, propensity score matching, MIMIC-IV

## Abstract

**Background:**

While early enteral nutrition (EN) is recommended for critically ill patients, its specific impact on mechanically ventilated chronic obstructive pulmonary disease (COPD) patients remains uncertain.

**Methods:**

We analyzed data extracted from the MIMIC-IV 3.0 database, focusing on patients with COPD who received invasive mechanical ventilation. The cohort was stratified into two groups: the early EN group (EEN, EN initiated within 48 h of ICU admission), and the delayed EN group (DEN, EN initiated after 48 h of ICU admission). Propensity score matching (PSM) was employed to balance baseline characteristics between the groups, enabling a comparative analysis of clinical outcomes.

**Results:**

Among 1,052 patients, 513 (48.76%) were in the early EN group and 539 (51.24%) were in the delayed EN group. After PSM, no statistically significant differences were observed in 28-day mortality (30.51% vs. 32.82%, *p* = 0.488), ICU mortality (17.18% vs. 21.28%, *p* = 0.146), or 60-day mortality (38.21% vs. 39.74%, *p* = 0.660). Similarly, the incidence of ventilator-associated pneumonia (VAP) did not differ significantly between the EEN and DEN groups (20.77% vs. 23.33%, *p* = 0.388). However, the EEN group exhibited a significantly shorter duration of mechanical ventilation (127.50 vs. 137.94 h, *p* = 0.023), reduced ICU length of stay (9.08 vs. 10.07 days, *p* < 0.01) and total hospitalization (14.64 vs. 16.63 days, *p* = 0.001). Additionally, subgroup analysis revealed that EEN significantly reduced 28-day mortality in patients with PaO_2_/FiO_2_ >200 (OR = 0.626, 95% CI: 0.414–0.943; *p* = 0.026).

**Conclusion:**

Although early EN did not significantly improve overall mortality, it effectively decreased ventilation duration and hospital stays and demonstrated potential survival benefits for patients with better oxygenation. These findings provide critical evidence for optimizing nutritional support strategies in mechanically ventilated COPD patients.

## 1 Introduction

Chronic obstructive pulmonary disease (COPD) is a prevalent chronic respiratory disorder characterized by progressive airflow limitation and recurrent lower respiratory tract infections (1, 2). According to the 2019 Global Burden of Disease Study, COPD represents a significant global health burden, with 212.3 million reported cases and 3.3 million annual deaths, ranking as the third leading cause of mortality worldwide (3). Predictive modeling studies project a 23% increase in COPD prevalence among individuals aged 25 years and older from 2020 to 2050, with the global patient population expected to approach 600 million by 2050 (4). This epidemiological trend underscores the critical need to identify modifiable risk factors that may mitigate COPD-related morbidity and mortality.

Malnutrition has emerged as a significant modifiable risk factor in COPD management (5). Extensive research demonstrates that COPD patients frequently exhibit compromised nutritional status, with advanced-stage patients showing marked reductions in body mass index (BMI), fat-free mass, handgrip strength, and respiratory and skeletal muscle function (6, 7). The pathophysiological consequences of malnutrition in COPD patients primarily manifest as reduced respiratory muscle mass, particularly affecting the diaphragm, which impairs respiratory muscle function, ventilatory capacity, and pulmonary defense mechanisms, ultimately leading to diminished lung function (8). For mechanically ventilated patients, diaphragmatic function represents a critical determinant of successful ventilator weaning. Furthermore, clinical studies have established that malnutrition significantly reduces quality of life in COPD patients, predisposes to acute respiratory failure, and increases the incidence of adverse respiratory and cardiovascular events (9).

As a potentially modifiable independent risk factor, malnutrition management through targeted nutritional interventions has demonstrated significant therapeutic potential in COPD care. Nutritional rehabilitation in COPD patients enhances immune function through improved neutrophil activity and complement system response, thereby augmenting host defense mechanisms against infections (10). Consequently, nutritional support has become an integral component of comprehensive COPD management and a critical factor in facilitating successful ventilator weaning (11). The timing of nutritional intervention initiation is particularly crucial for optimizing clinical outcomes in critically ill patients (12). Emerging evidence suggests that early standardized enteral nutrition may prevent acute muscle loss and intensive care unit-acquired weakness (ICU-AW) in patients experiencing acute exacerbations of COPD (AECOPD) (13). However, the prognostic implications of early enteral nutrition in mechanically ventilated COPD patients remain incompletely characterized and warrant further investigation. This study aims to evaluate the efficacy of early enteral nutrition (EN) in improving clinical outcomes among mechanically ventilated COPD patients.

## 2 Materials and methods

### 2.1 Overview

This investigation constitutes a retrospective observational analysis utilizing the Medical Information Mart for Intensive Care IV (MIMIC-IV) database (version 3.0, updated July 23, 2024). MIMIC-IV represents a comprehensive, single-center repository encompassing clinical data from patients admitted to the intensive care unit (ICU) of a tertiary care hospital in Boston, Massachusetts, USA. The database comprises hospitalization records of 94,458 adult patients (≥18 years) admitted to the ICU between 2008 and 2022.

All data within the database have undergone rigorous de-identification procedures, ensuring the anonymity of individual patients. Consequently, this study does not qualify as human subjects research under current regulatory guidelines, and the use of de-identified health information obviates the requirement for patient consent. The development and maintenance of the MIMIC-IV 3.0 database received ethical approval from the Institutional Review Boards of the Massachusetts Institute of Technology (MIT, Cambridge, Massachusetts) and Beth Israel Deaconess Medical Center (BIDMC). Author Lamei Ouyang obtained authorized access to the MIMIC-IV 3.0 database following successful completion of the requisite Human Subject Research course (certification number: 64058594).

### 2.2 Participant selection

**Figure 1:**
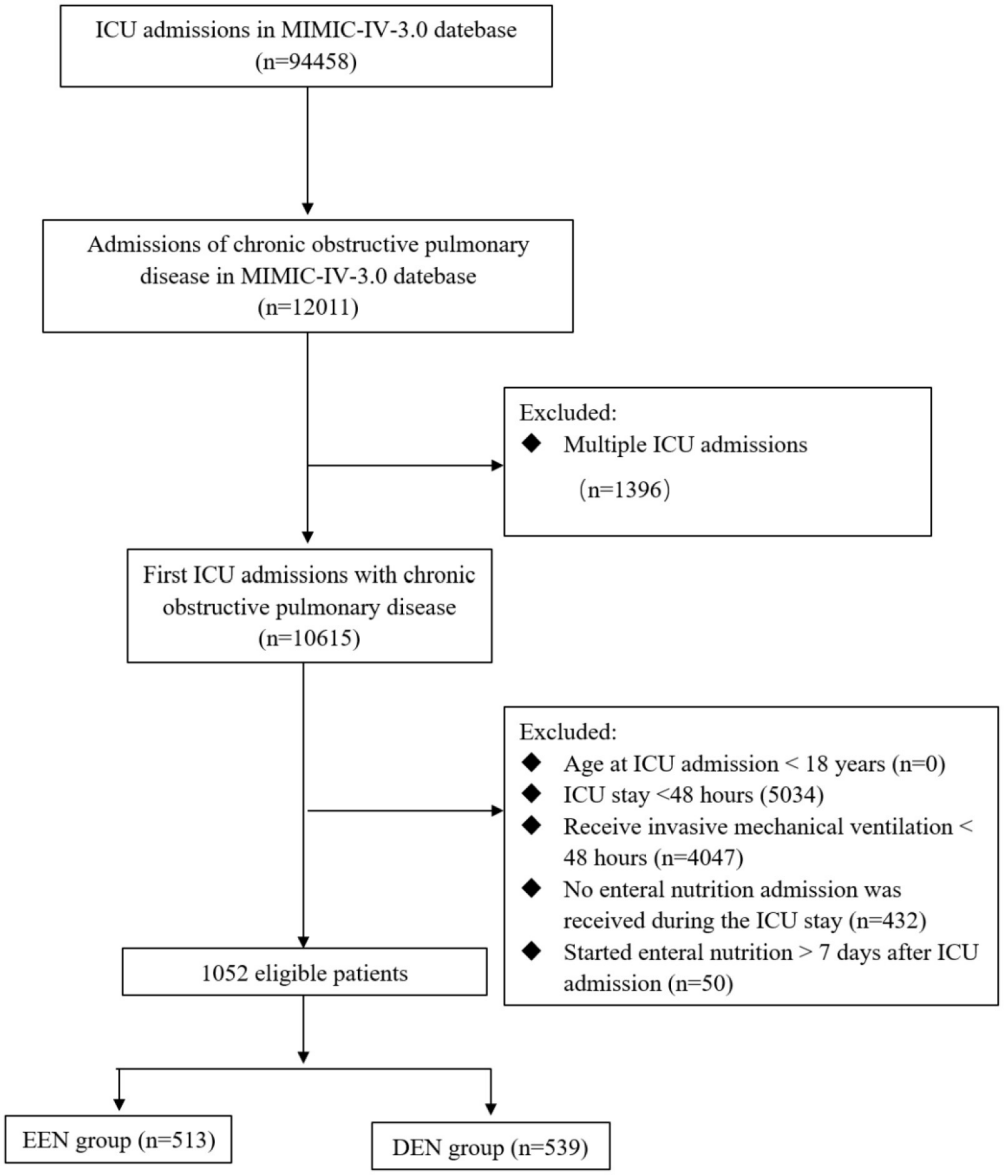
Flow chart of participant selection. MIMIC-IV, medical information mart for intensive care IV; ICU, intensive care unit; EEN, early enteral nutrition; DEN, delayed enteral nutrition.

This study enrolled patients aged ≥18 years diagnosed with COPD according to established diagnostic criteria (14), who underwent invasive mechanical ventilation and received EN during their ICU stay. The analysis was restricted to patients experiencing their first ICU admission. Exclusion criteria comprised: (1) age <18 years at ICU admission; (2) ICU stay duration <48 h; (3) receive invasive mechanical ventilation <48 h; (4) no enteral nutrition admission was received during the ICU stay; and (5) started enteral nutrition > 7 days after ICU admission (Figure 1 illustrates the patient flow diagram).

Comprehensive patient data were systematically collected, encompassing demographic characteristics (age, sex, BMI, and ethnicity) and physiological parameters recorded within the initial 24 h of ICU admission, including heart rate, respiratory rate, body temperature, mean arterial pressure, blood glucose levels, and 24-h urine output. Laboratory analyses included arterial blood gas parameters (pH, partial pressure of oxygen [PO_2_], partial pressure of carbon dioxide [PCO_2_], PaO_2_/FiO_2_ ratio, lactate), hematological indices (white blood cell count [WBC], hemoglobin, platelet count), coagulation profile (activated partial thromboplastin time [APTT]), and biochemical parameters (creatinine, blood urea nitrogen, and electrolyte levels [chloride, calcium, potassium, sodium]). For variables with multiple measurements within 24 h, mean values were calculated.

Interventional data captured within the first 24 h included administration of vasopressors, implementation of continuous renal replacement therapy, invasive arterial pressure monitoring, and placement of peripherally inserted central catheters. Disease severity was quantified using validated scoring systems: sequential Organ Failure Assessment (SOFA), Glasgow Coma Scale (GCS), Acute Physiology Score III (APS III), and Charlson Comorbidity Index (CCI). Documented comorbidities included congestive heart failure, cerebrovascular disease, liver disease, diabetes mellitus, renal disease, and cancer.

### 2.3 Grouping

In accordance with the most recent clinical guidelines for nutritional support in critically ill patients, participants were stratified into two distinct cohorts: the early enteral nutrition (EEN) group, defined by the initiation of EN within 48 h following ICU admission, and the delayed enteral nutrition (DEN) group, characterized by EN initiation beyond 48 h post-ICU admission (15).

### 2.4 Statistical analysis

The normality of continuous variables was assessed using both the Kolmogorov-Smirnov and Shapiro-Wilk tests. As all continuous variables exhibited non-normal distributions, they were expressed as medians with interquartile ranges (IQRs), and comparisons between the two groups were performed using the Mann-Whitney U test. Categorical variables were presented as proportions and analyzed using the chi-square test.

To mitigate potential confounding factors, propensity score matching (PSM) was employed before comparing outcomes between the EEN and DEN groups (16). A propensity score was calculated for each patient using a logistic regression model including 39 potential baseline prognostic and risk factors in Table 1, and then individuals were matched using a 1:1 nearest neighbor matching approach with a caliper width of 0.2 standard deviations of the propensity score logit. Post-matching balance was evaluated using standardized mean differences (SMDs), with an SMD threshold of >0.1 indicating imbalance (17, 18). All PSM analyses were conducted using R software (version 4.4.1).

**Table 1:** Clinical characteristics of patients before and after propensity score matching.

**Variables**	**Before PSM**			**After PSM**		
	**Early EN (*****n*** = **513)**	**Delayed EN (*****n*** = **539)**	***p*** **-value**	**Early EN (*****n*** = **390)**	**Delayed EN (*****n*** = **390)**	***p*** **-value**
Age (years)	70.28 [62.06, 78.05]	71.22 [62.91, 77.90]	0.834	70.02 [62.03, 77.76]	70.75 [62.86, 77.39]	0.873
Male (%)	281 (54.78)	311 (57.70)	0.339	220 (56.41)	222 (56.92)	0.885
BMI (kg/m^2^)	27.58 [23.37, 34.96]	28.28 [23.73, 34.40]	0.367	27.16 [23.11, 35.23]	27.97 [23.32, 33.81]	0.712
**Race (%)**			0.034			0.638
White	329 (64.13)	366 (67.90)		254 (65.13)	257 (65.90)	
Black	54 (10.53)	33 (6.12)		34 (8.72)	27 (6.92)	
Other or unknown	130 (25.34)	140 (25.97)		102 (26.15)	106 (27.18)	
**Vital indicators**						
HR (bpm)	84.16 [73.38, 95.80]	86.46 [74.15, 99.16]	0.018	85.35 [75.06, 97.01]	85.52 [73.41, 98.28]	0.961
RR (bpm)	20.58 [17.96, 23.15]	20.00 [17.80, 22.80]	0.273	20.55 [17.93, 23.45]	20.17 [17.89, 22.82]	0.558
Temperature (°C)	36.98 [36.71, 37.28]	36.88 [36.61, 37.26]	0.028	36.97 [36.69, 37.31]	36.89 [36.61, 37.29]	0.219
MAP(mmHg)	75.04 [69.96, 81.15]	74.56 [69.10, 80.98]	0.374	75.50 [70.30, 80.76]	74.14 [68.95, 81.20]	0.334
Glucose (mg/dL)	138.29 [112.75, 170.89]	140.75 [117.67, 171.12]	0.282	140.46 [113.69, 176.96]	138.22 [117.06, 169.23]	0.685
First-day Urine Output (mL)	1,290.00 [831.00, 2,010.00]	1,230.00 [730.50, 1,882.00]	0.169	1,258.50 [765.00, 1,950.00]	1,250.00 [775.00, 1,932.75]	0.969
**Laboratory indicators**						
PH	7.35 [7.30, 7.41]	7.34 [7.28, 7.40]	0.002	7.34 [7.29, 7.39]	7.35 [7.29, 7.40]	0.975
PO_2_ (mm Hg)	107.00 [86.00, 145.00]	121.00 [92.00, 171.82]	<0.001	111.30 [87.72, 154.75]	114.75 [90.00, 163.20]	0.317
PCO_2_ (mm Hg)	48.00 [41.50, 57.25]	44.29 [39.00, 51.39]	<0.001	46.88 [41.00, 55.42]	45.81 [39.89, 54.77]	0.140
Pao_2_/Fio_2_ (P/F, mmHg)	205.34 [151.17, 278.00]	217.74 [157.91, 288.96]	0.110	211.33 [152.13, 285.50]	217.48 [156.50, 286.88]	0.457
Lactate (mmol/L)	1.36 [1.00, 1.97]	1.60 [1.10, 2.50]	<0.001	1.40 [1.05, 2.17]	1.47 [1.10, 2.30]	0.18
WBC (×10∧9/L)	11.70 [8.35, 15.93]	12.60 [8.96, 17.18]	0.020	11.84 [8.65, 16.34]	12.25 [8.88, 16.26]	0.354
Hemoglobin (g/dL)	10.30 [8.90, 11.95]	10.37 [9.00, 11.79]	0.996	10.44 [9.03, 12.20]	10.43 [9.00, 11.85]	0.433
Platelets (×10∧9/L)	206.50 [149.50, 281.25]	191.00 [134.28, 258.54]	0.007	202.00 [146.58, 267.75]	197.25 [137.62, 271.62]	0.454
APTT	31.80 [27.50, 39.25]	33.50 [28.59, 43.74]	0.001	32.40 [27.80, 41.04]	33.36 [28.40, 42.82]	0.158
BUN (mg/dL)	25.67 [17.80, 44.00]	25.20 [17.41, 40.90]	0.146	25.09 [17.27, 40.00]	26.62 [18.00, 44.19]	0.524
Creatinine (mg/dL)	1.10 [0.70, 1.87]	1.17 [0.80, 1.83]	0.063	1.10 [0.73, 1.90]	1.15 [0.80, 1.83]	0.349
Calcium (mg/dL)	8.35 [7.85, 8.80]	8.17 [7.68, 8.66]	<0.001	8.27 [7.77, 8.70]	8.24 [7.75, 8.76]	0.807
Chloride (mmol/L)	102.00 [97.50, 106.00]	103.60 [99.50, 107.00]	0.001	102.33 [98.35, 106.67]	103.00 [99.00, 106.32]	0.679
Sodium (mmol/L)	139.67 [136.33, 142.50]	139.00 [136.00, 141.50]	0.005	139.33 [135.67, 142.24]	139.00 [136.43, 142.00]	0.674
Potassium (mmol/L)	4.23 [3.87, 4.77]	4.34 [3.90, 4.78]	0.164	4.25 [3.90, 4.80]	4.35 [3.90, 4.70]	0.841
**Medications and interventions**						
Vasoactive agent (%)	353 (68.81)	451 (83.67)	<0.001	300 (76.92)	307 (78.72)	0.546
Continuous renal replacement therapy (%)	51 (9.94)	89 (16.51)	0.002	46 (11.79)	52 (13.33)	0.517
Invasive arterial pressure monitoring (%)	322 (62.77)	428 (79.41)	<0.001	276 (70.77)	287 (73.59)	0.380
Peripherally inserted central catheter (%)	249 (48.54)	245 (45.45)	0.317	175 (44.87)	184 (47.18)	0.518
**Disease severity scoring system**						
SOFA	7.00 [5.00, 10.00]	7.00 [5.00, 10.00]	0.434	7.00 [5.00, 10.00]	7.00 [5.00, 10.00]	0.882
GCS	15.00 [13.00, 15.00]	15.00 [14.00, 15.00]	0.834	15.00 [13.00, 15.00]	15.00 [14.00, 15.00]	0.822
APS III	54.00 [42.00, 68.00]	55.00 [45.00, 71.00]	0.038	56.00 [43.00, 69.00]	54.50 [44.00, 67.75]	0.857
CCI	6.00 [5.00, 8.00]	6.00 [5.00, 8.00]	0.018	6.00 [5.00, 8.00]	6.00 [5.00, 8.00]	0.359
**Comorbidities**						
Congestive heart failure (%)	236 (46.00)	271 (50.28)	0.166	185 (47.44)	190 (48.72)	0.720
Cerebrovascular_disease (%)	82 (15.98)	78 (14.47)	0.495	62 (15.90)	62 (15.90)	1.000
Liver disease (%)	65 (12.67)	90 (16.70)	0.065	49 (12.56)	59 (15.13)	0.300
Diabetes (%)	180 (35.09)	191 (35.44)	0.906	141 (36.15)	138 (35.38)	0.823
Renal disease (%)	136 (26.51)	136 (25.23)	0.636	100 (25.64)	103 (26.41)	0.807
Cancer (%)	61 (11.89)	79 (14.66)	0.187	54 (13.85)	50 (12.82)	0.674

PSM, propensity score matching; EN, enteral nutrition; BMI, body mass index; HR, heart rate; RR, respiratory rate; MAP, mean arterial pressure; PH, potential of hydrogen; PO_2_, partial pressure of oxygen; PCO_2_, partial pressure of carbon dioxide; WBC, white blood cell; APTT, activated partial thromboplastin time; BUN, blood urea nitrogen; SOFA, sequential organ failure assessment; GCA, glasgow ccoma scale; APS III, acute physiology score III; CCI, Charlson comorbidity index.

The primary outcome was 28-day mortality. Secondary outcomes included 60-day mortality, ICU mortality, the incidence of ventilator-associated pneumonia (VAP), hospital length of stay (LOS hospital), ICU length of stay (LOS ICU), and duration of invasive mechanical ventilation. For both primary and secondary endpoints, categorical variables were compared between groups using the Chi-square test, while continuous variables were compared using the Mann-Whitney U test. Subsequently, the 28-day survival, ICU survival and 60-day survival were estimated using Kaplan-Meier (K-M) survival curves, and the hazard ratio between two groups was estimated using the Cox proportional hazards model. Subgroup analyses were performed to identify populations potentially benefiting from EEN, stratified by age, sex, lactate level, BMI, and PaO_2_/FiO_2_ ratio. The associations between EEN and 28-day mortality were quantified using univariate logistic analysis, with results visualized in forest plots. Furthermore, to delineate independent associations, we performed distinct sensitivity analyses by excluding patients with cancer diagnoses, non-white individuals, or patients not receiving vasoactive agents. These analyses aimed to examine the reliability and applicability of our results across diverse patient groups and clinical scenarios. Variables exceeding 30% missingness were excluded from the analysis. Then, missing data for covariates were addressed using multiple imputation via the MICE package in R (Supplementary Figure S1). The primary analysis model was applied to each of 20 imputed dataset, and estimates were pooled using Rubin's rules to derive final parameter estimates with standard errors accounting for missing data uncertainty. All statistical analyses were conducted in R (version 4.4.1), with a two-sided *p*-value < 0.05 considered statistically significant.

## 3 Results

### 3.1 Demographic data and baseline characteristics

The study cohort consisted of 1,052 patients, as depicted in Figure 1. Among these, 513 patients (48.76%) were classified into the EEN group, defined by the initiation of EN within 48 h of ICU admission, while 539 patients (51.24%) were assigned to the DEN group, characterized by EN initiation beyond 48 h of ICU admission. The demographic and clinical characteristics of both groups are presented in Table 1. Notably, during the first 24 h of ICU admission, the DEN group demonstrated significantly higher lactate levels (1.60 [1.10, 2.50] vs. 1.36 [1.00, 1.97]; *p* < 0.001) and elevated disease severity scores, including the Acute Physiology Score III (APS III) (55.00 [45.00, 71.00] vs. 54.00 [42.00, 68.00]; *p* = 0.038) and Charlson Comorbidity Index (CCI) (6.00 [5.00, 8.00] vs. 6.00 [5.00, 8.00]; *p* = 0.018). Additionally, the DEN group exhibited a higher prevalence of vasopressor support (451 [83.67%] vs. 353 [68.81%]; *p* < 0.001), invasive arterial pressure monitoring (428 [79.41%] vs. 322 [62.77%]; *p* < 0.001), and continuous renal replacement therapy (89 [16.51%] vs. 51 [9.94%]; *p* = 0.002). After PSM, there was no significant differences in all these baseline characteristics, with all SMDs < 0.10 (Supplementary Table S1).

### 3.2 Comparison of primary outcomes before and after propensity score matching

**Figure 2:**
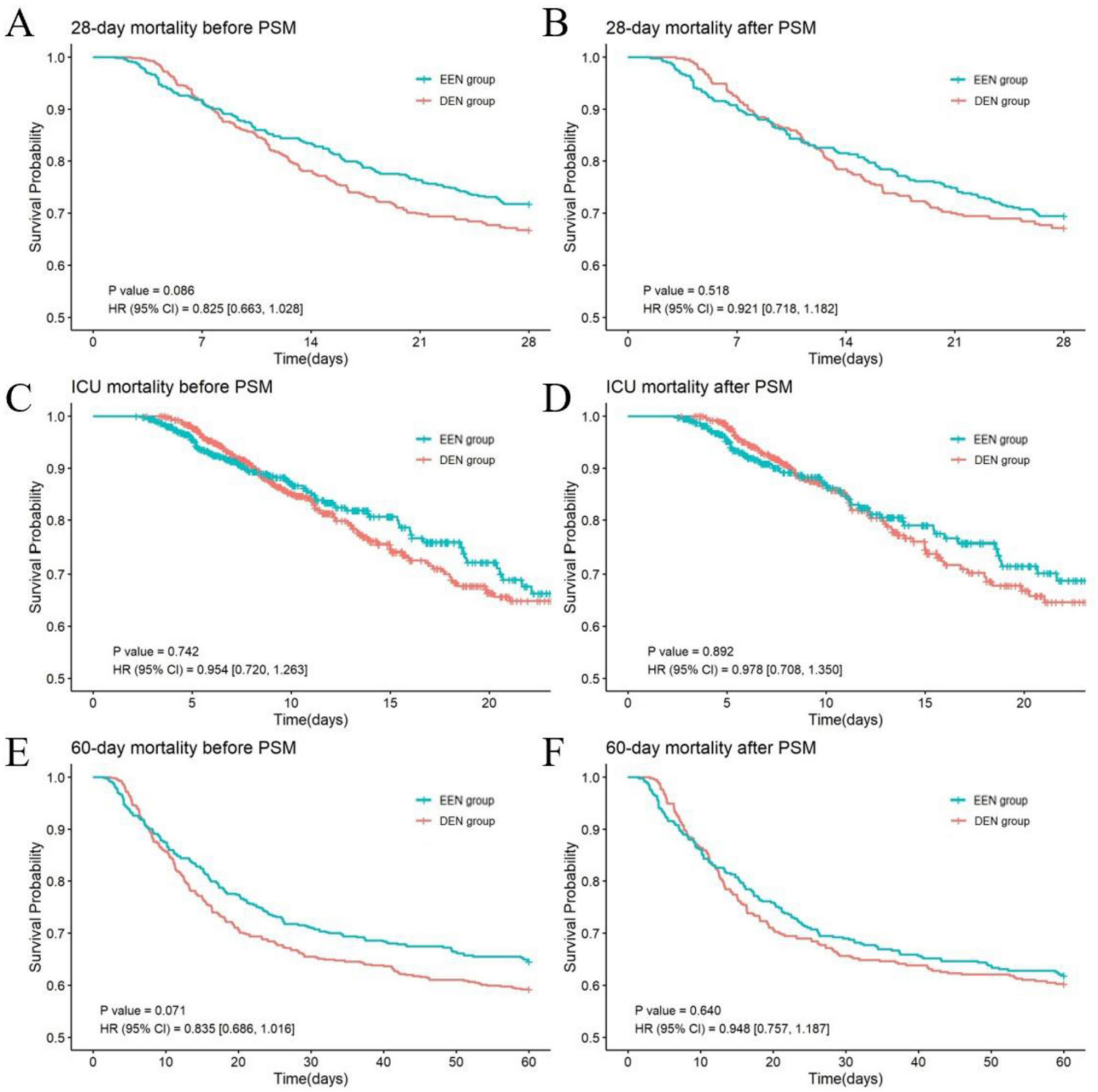
Kaplan–Meier survival curves of the two groups at 28 days **(A, B)**, ICU **(C, D)** and 60 days **(E, F)** before and after propensity score matching.

Prior to PSM, no statistically significant difference in 28-day mortality was observed between the EEN group and the DEN group (145 [28.27%] vs. 179 [33.21%]; *p* = 0.082) (Table 2). The univariate Kaplan–Meier survival curve for 28 days further confirmed the absence of a significant difference in survival time between the EEN and DEN groups (HR = 0.825, 95% CI: 0.663–1.028; *p* = 0.086) (Figure 2A). Post-matching analysis revealed a 2.31% reduction in 28-day mortality in the EEN group compared to the DEN group; however, this difference did not reach statistical significance (119 [30.51%] vs. 128 [32.82%]; *p* = 0.488). The 28-day Kaplan–Meier curve after propensity matching was consistent with the result (HR = 0.921, 95% CI: 0.718–1.182; *p* = 0.518) (Figure 2B).

**Table 2:** Primary and secondary outcomes.

**Outcomes**	**Before PSM**			**After PSM**		
	**Early EN (*****n*** = **513)**	**Delayed EN (*****n*** = **539)**	***p*** **-value**	**Early EN (*****n*** = **390)**	**Delayed EN (*****n*** = **390)**	***p*** **-value**
28-day mortality (%)	145 (28.27)	179 (33.21)	0.082	119 (30.51)	128 (32.82)	0.488
ICU mortality (%)	83 (16.18)	119 (22.08)	0.015	67 (17.18)	83 (21.28)	0.146
60-day mortality (%)	182 (35.48)	220 (40.82)	0.075	149 (38.21)	155 (39.74)	0.660
VAP(%)	98 (19.10)	121 (22.45)	0.182	81 (20.77)	91 (23.33)	0.388
LOS ICU (days)	8.17 [5.17, 14.45]	10.65 [7.44, 17.20]	<0.001	9.08 [5.39, 15.35]	10.07 [7.24, 16.11]	<0.001
LOS hospital (days)	13.58 [8.35, 20.37]	16.74 [10.87, 25.86]	<0.001	14.64 [8.97, 21.46]	16.63 [10.68, 25.58]	0.001
Invasive mechanical ventilation (hours)	116.90 [79.58, 233.68]	144.88 [95.47, 260.12]	<0.001	127.50 [80.55, 247.23]	137.94 [93.05, 266.69]	0.023

ICU, intensive care unit; VAP, ventilator-associated pneumonia; LOS, length of stay.

### 3.3 Comparison of secondary outcomes before and after propensity score matching

Prior to PSM, the EEN group exhibited significantly lower ICU mortality compared to the DEN group (83 [16.18%] vs. 119 [22.08%]; *p* = 0.015), whereas no significant difference was observed in 60-day mortality (182 [35.48%] vs. 220 [40.82%]; *p* = 0.075) (Table 2). Univariate Kaplan-Meier analysis for ICU survival (HR = 0.954, 95% CI: 0.720–1.263; *p* = 0.742) (Figure 2C) and 60-day survival (HR = 0.835, 95% CI: 0.686–1.016; *p* = 0.071) (Figure 2E) demonstrated no significant intergroup difference. The incidence of VAP did not differ significantly between the EEN and DEN groups (98 [19.10%] vs. 121 [22.45%]; *p* = 0.182). However, the EEN group showed significantly shorter total hospital stays (13.58 [8.35, 20.37] vs. 16.74 [10.87, 25.86] days; *p* < 0.001), reduced ICU stays (8.17 [5.17, 14.45] vs. 10.65 [7.44, 17.20] days; *p* < 0.001), and decreased duration of invasive mechanical ventilation (116.90 [79.58, 233.68] vs. 144.88 [95.47, 260.12] hours; *p* < 0.001). Following PSM, no significant differences were observed in ICU mortality (67 [17.18%] vs. 83 [21.28%]; *p* = 0.146) or 60-day mortality (149 [38.21%] vs. 155 [39.74%]; *p* = 0.660). The ICU Kaplan–Meier curve (HR = 0.978, 95% CI: 0.708–1.350; *p* = 0.892) (Figure 2D) and 60-day Kaplan–Meier curve (HR = 0.948, 95% CI: 0.757–1.187; *p* = 0.640) (Figure 2F) post-propensity matching echoed the propensity-matched result. VAP incidence remained comparable between groups (81 [20.77%] vs. 91 [23.33%]; *p* = 0.388). Nonetheless, the EEN group maintained significantly shorter total hospital stays (14.64 [8.97, 21.46] vs. 16.63 [10.68, 25.58] days; *p* = 0.001), reduced ICU stays (9.08 [5.39, 15.35] vs. 10.07 [7.24, 16.11] days; *p* < 0.001), and decreased duration of invasive mechanical ventilation (127.50 [80.55, 247.23] vs. 137.94 [93.05, 266.69] hours; *p* = 0.023).

### 3.4 Additional analyses

**Figure 3:**
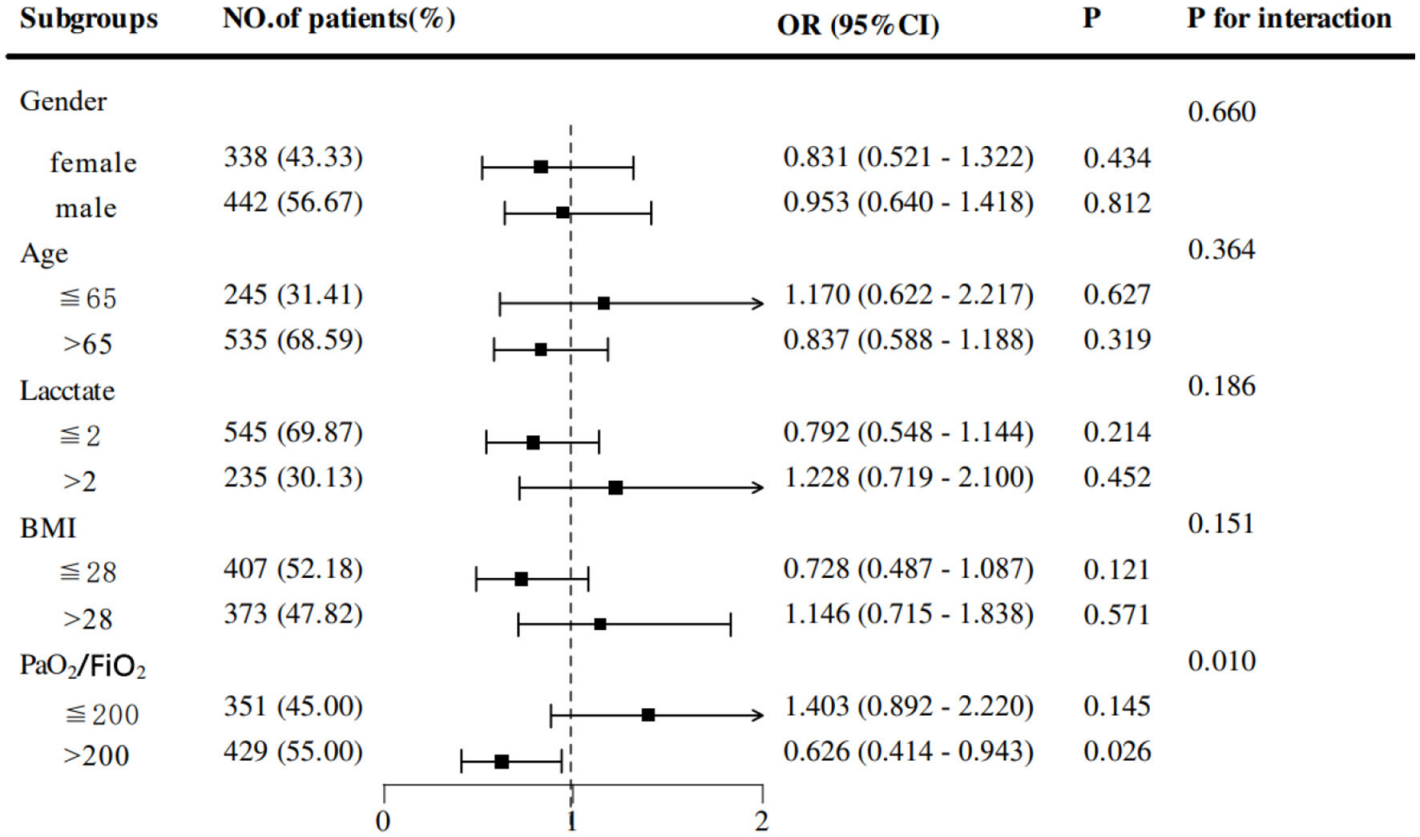
Subgroup analyses to identify the specific benefit population.

**Figure 4:**
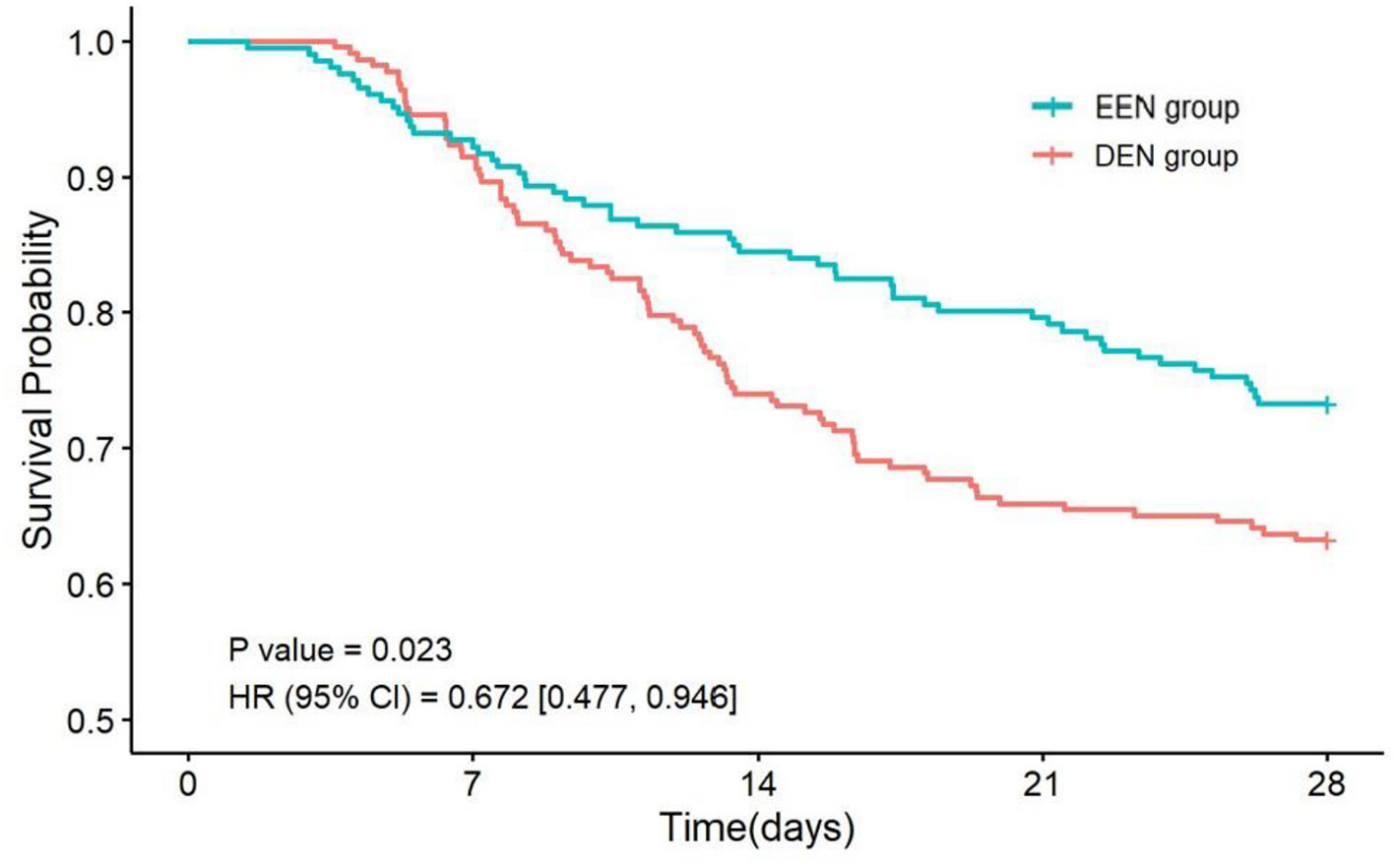
The Kaplan–Meier survival curve adjusted for the subgroup with PaO_2_/FiO_2_>200.

Subgroup analyses based on propensity-matched data were conducted to explore the association between early EN and 28-day mortality across subgroups of COPD patients. Stratifications included sex, age, lactate levels, BMI, and PaO_2_/FiO_2_ ratio. Results revealed that EEN was significantly associated with a reduction in 28-day mortality in patients with PaO_2_/FiO_2_ > 200 (OR = 0.626, 95% CI: 0.414–0.943; *p* = 0.026) (Figure 3). Adjusted Kaplan-Meier survival curves from Cox regression analysis are presented in Figure 4.

### 3.5 Sensitivity analysis

There were 1,052 patients in the entire cohort. We performed further sensitivity analyses after excluding 140 patients with cancer diagnoses (HR: 0.871; 95% CI: 0.665–1.140; *p* = 0.314), 357 non-white individuals (HR: 0.842; 95% CI: 0.615–1.153; *p* = 0.284), and 248 patients not receiving vasoactive agents (HR: 0.959; 95% CI: 0.739–1.246; *p* = 0.756), respectively, and the results were consistent with the primary outcome (Table 3).

**Table 3:** Sensitivity analysis of the relationship between early enteral nutrition and 28 day mortality.

**Sensitivity**	**Matching**	**28 day mortality, n/N (%)**				**Correlation analysis**		
		**Total**	**EEN**	**DEN**	***P***	**HR**	**95%CI**	***P***
Model 1 (*n* = 912)	Before PSM	257/912 (28.18%)	116/460 (25.66%)	141/452 (30.65%)	0.094	0.871^a^	0.665-1.140	0.314
	After PSM	191/650 (29.38%)	90/325 (27.69%)	101/325 (31.08)	0.344	0.844^b^	0.629-1.132	0.257
Model 2 (*n* = 695)	Before PSM	204/695 (29.35%)	91/329 (27.66%)	113/366 (30.87%)	0.353	0.842^a^	0.615-1.153	0.284
	After PSM	140/462 (30.30%)	67/231 (29.00%)	73/231 (31.60%)	0.544	0.898^b^	0.631-1.278	0.551
Model 3 (*n* = 804)	Before PSM	277/804 (34.45%)	120/353 (33.99%)	157/451 (34.81%)	0.809	0.959^a^	0.739-1.246	0.756
	After PSM	211/602 (35.05%)	105/301 (34.88%)	106/301 (35.22%)	0.932	0.983^b^	0.739-1.306	0.934

Model 1: Excluded participants with cancer; Model 2: Excluded non-white participants; Model 3: Excluded participants without vasoactive agent.

a HR, from a multivariable Cox proportional model adjusted for all covariates in Table 1.

b HR, from a multivariable Cox proportional hazards model with the same strata and covariates, with additional adjustment for the propensity score.

## 4 Discussion

This retrospective cohort study analyzed 1,052 mechanically ventilated patients with COPD from the MIMIC-IV database to evaluate the effects of early EN vs. delayed EN on clinical outcomes. The analysis revealed that while early EN did not demonstrate statistically significant reductions in mortality rates, it was associated with significantly shorter durations of invasive mechanical ventilation, reduced ICU and hospital lengths of stay, and improved survival trends in specific patient subgroups. These findings underscore the potential clinical benefits of timely nutritional intervention in this critically ill population.

The observed mortality outcomes align with previous studies, suggesting that early EN may not substantially improve survival rates in this patient population (19–21). This phenomenon may be attributed to the complex pathophysiology of COPD and the frequent occurrence of multi-organ dysfunction in critically ill patients. While early nutritional support has been shown to preserve gut barrier function and mitigate infection risks, its impact on mortality may be modulated by multiple confounding factors, including disease severity, comorbid conditions, and the patient's overall metabolic state (22–25). Furthermore, the significant reduction in both ICU and hospital lengths of stay associated with early EN corroborates findings from previous investigations (19, 20, 24). This effect may be mediated through improved metabolic homeostasis, reduced incidence of infectious complications, and enhanced recovery processes, thereby facilitating more efficient patient rehabilitation.

The present study demonstrates that early EN significantly reduces the duration of mechanical ventilation, a finding that aligns with previous investigations (26–28). This effect may be attributed to multiple mechanisms through which early nutritional support improves clinical outcomes. First, early EN helps maintain respiratory muscle function. Patients with COPD often suffer from respiratory muscle weakness, and prolonged mechanical ventilation may exacerbate disuse atrophy of these muscles (29–31). Lower respiratory muscle strength plays a significant role in COPD and is associated with an increased risk of exacerbation. Respiratory muscle function could serve as a marker of disease status and early prognosis in COPD (29). Early nutritional support provides adequate energy and protein, helping to maintain respiratory muscle strength and endurance, thereby reducing dependence on mechanical ventilation (13). Studies have shown that malnutrition is a significant factor contributing to respiratory muscle weakness, and early EN can improve nutritional status, subsequently reducing the duration of mechanical ventilation (26–28). Second, early EN may shorten the duration of mechanical ventilation by reducing infectious complications (15, 32–34). Mechanically ventilated patients are prone to VAP, and early EN helps maintain gut barrier function, reducing the risk of bacterial translocation and systemic infections. Several studies have shown that early EN can lower the incidence of VAP, thereby reducing the duration of mechanical ventilation (19, 35, 36). Additionally, early nutritional support can enhance immune function, further reducing infection risks. Third, early EN may reduce the duration of mechanical ventilation by improving metabolic status and reducing inflammatory responses (37–40). Critically ill patients often experience metabolic disturbances and systemic inflammation, which can prolong mechanical ventilation. Early nutritional support can modulate metabolic status and reduce the release of inflammatory mediators, thereby promoting recovery. Studies have shown that early EN can lower levels of inflammatory markers such as C-reactive protein (CRP) and interleukin-6 (IL-6), subsequently reducing the duration of mechanical ventilation (41, 42).

Subgroup analyses revealed that EEN significantly reduced 28-day mortality in patients with a PaO_2_/FiO_2_ ratio > 200. This subgroup likely represents patients with less severe hypoxemia and preserved pulmonary function, where early nutrition could synergize with better baseline oxygenation to optimize recovery (43). The interaction between adequate oxygenation and metabolic support may enhance cellular repair processes and reduce oxidative stress, thereby improving survival. Conversely, in patients with severe hypoxemia (PaO_2_/FiO_2_ ≤ 200), the benefits of EEN might be overshadowed by overwhelming physiological derangements, necessitating more aggressive interventions. These findings advocate for personalized nutritional strategies, prioritizing EEN in COPD patients with better oxygenation to optimize clinical outcomes. Future studies should validate this oxygenation threshold and investigate synergistic effects of combined oxygen therapy and nutritional support.

This study has several limitations. Despite a sample size of 1,052 participants, this study remained underpowered to detect very small effects. Definitive conclusions regarding such minimal effect magnitudes would require larger cohorts. As a retrospective analysis, unmeasured confounders (e.g., variations in clinician practices, unrecorded comorbidities) may influence outcomes. Future prospective, multi-center randomized controlled trials are warranted to validate these findings and further elucidate optimal nutritional strategies in this high-risk cohort. Despite rigorous PSM, residual bias cannot be entirely excluded. The single-center design and reliance on the MIMIC-IV database limit generalizability to other settings. Additionally, the definition of EEN as initiation within 48 h may not reflect optimal timing, as some studies recommend even earlier initiation (44, 45).

In conclusion, our retrospective study demonstrates that early EN, while not significantly reducing 28-day mortality in mechanically ventilated COPD patients, significantly shortens mechanical ventilation duration, ICU/hospital stays, and lowers mortality risk in the subgroup with PaO_2_/FiO_2_ >200. Early EN holds clinical value by enhancing metabolic support and reducing complications, thereby improving recovery and conserving healthcare resources.

## Data Availability

The raw data supporting the conclusions of this article will be made available by the authors, without undue reservation.
