# Supplementary material for: Impact of early enteral nutrition on the prognosis of mechanically ventilated patients with chronic obstructive pulmonary disease: a retrospective cohort study based on the MIMIC-IV Database
Source: Front Nutr. 2025 Aug 13;12:1620011. doi: 10.3389/fnut.2025.1620011 (PMC12380561; doi:10.3389/fnut.2025.1620011)
Supplement: Supplementary file 1 [file Data_Sheet_1.docx]

**Supplementary Table S1**

|  | **Before PSM** | | | | **After PSM** | | | |
| --- | --- | --- | --- | --- | --- | --- | --- | --- |
| **Variables** | **Early EN（n=513）** | **Delayed EN（n=539）** | ***p*-value** | **SMD** | **Early EN（n=390）** | **Delayed EN（n=390）** | ***p*-value** | **SMD** |
| **Age(years)** | 70.28 [62.06, 78.05] | 71.22 [62.91, 77.90] | 0.834 | 0.005 | 70.02 [62.03, 77.76] | 70.75 [62.86, 77.39] | 0.873 | 0.004 |
| **Male(%)** | 281 (54.78) | 311 (57.70) | 0.339 | 0.059 | 220 (56.41) | 222 (56.92) | 0.885 | 0.01 |
| **BMI(kg/m2)** | 27.58 [23.37, 34.96] | 28.28 [23.73, 34.40] | 0.367 | 0.047 | 27.16 [23.11, 35.23] | 27.97 [23.32, 33.81] | 0.712 | 0.015 |
| **Race (%)** |  | | 0.034 | 0.161 |  | | 0.638 | 0.068 |
| WHITE | 329 (64.13) | 366 (67.90) |  |  | 254 (65.13) | 257 (65.90) |  |  |
| BLACK | 54 (10.53) | 33 ( 6.12) |  |  | 34 ( 8.72) | 27 ( 6.92) |  |  |
| OTHER or UKNOWN | 130 (25.34) | 140 (25.97) |  |  | 102 (26.15) | 106 (27.18) |  |  |
| **Vital Indicators** | | | | | | | | |
| HR（bpm) | 84.16 [73.38, 95.80] | 86.46 [74.15, 99.16] | 0.018 | 0.158 | 85.35 [75.06, 97.01] | 85.52 [73.41, 98.28] | 0.961 | 0.006 |
| RR (bpm) | 20.58 [17.96, 23.15] | 20.00 [17.80, 22.80] | 0.273 | 0.058 | 20.55 [17.93, 23.45] | 20.17 [17.89, 22.82] | 0.558 | 0.024 |
| Temperature (°C) | 36.98 [36.71, 37.28] | 36.88 [36.61, 37.26] | 0.028 | 0.079 | 36.97 [36.69, 37.31] | 36.89 [36.61, 37.29] | 0.219 | 0.026 |
| MAP(mmHg) | 75.04 [69.96, 81.15] | 74.56 [69.10, 80.98] | 0.374 | 0.017 | 75.50 [70.30, 80.76] | 74.14 [68.95, 81.20] | 0.334 | 0.029 |
| Glucose (mg/dL) | 138.29 [112.75, 170.89] | 140.75 [117.67, 171.12] | 0.282 | 0.062 | 140.46 [113.69, 176.96] | 138.22 [117.06, 169.23] | 0.685 | 0.011 |
| First-day Urine Output (mL) | 1290.00 [831.00, 2010.00] | 1230.00 [730.50, 1882.00] | 0.169 | 0.086 | 1258.50 [765.00, 1950.00] | 1250.00 [775.00, 1932.75] | 0.969 | 0.036 |
| **Laboratory Indicators** | | | | | | | | |
| PH | 7.35 [7.30, 7.41] | 7.34 [7.28, 7.40] | 0.002 | 0.213 | 7.34 [7.29, 7.39] | 7.35 [7.29, 7.40] | 0.975 | 0.022 |
| PO_2_ (mm Hg) | 107.00 [86.00, 145.00] | 121.00 [92.00, 171.82] | <0.001 | 0.223 | 111.30 [87.72, 154.75] | 114.75 [90.00, 163.20] | 0.317 | 0.057 |
| PCO_2_ (mm Hg) | 48.00 [41.50, 57.25] | 44.29 [39.00, 51.39] | <0.001 | 0.288 | 46.88 [41.00, 55.42] | 45.81 [39.89, 54.77] | 0.140 | 0.043 |
| Pao_2_/Fio_2_（P/F, mmHg） | 205.34 [151.17, 278.00] | 217.74 [157.91, 288.96] | 0.110 | 0.096 | 211.33 [152.13, 285.50] | 217.48 [156.50, 286.88] | 0.457 | 0.038 |
| Lactate(mmol/L) | 1.36 [1.00, 1.97] | 1.60 [1.10, 2.50] | <0.001 | 0.252 | 1.40 [1.05, 2.17] | 1.47 [1.10, 2.30] | 0.18 | 0.063 |
| WBC (×10^9/L） | 11.70 [8.35, 15.93] | 12.60 [8.96, 17.18] | 0.020 | 0.14 | 11.84 [8.65, 16.34] | 12.25 [8.88, 16.26] | 0.354 | 0.074 |
| Hemoglobin (g/dL) | 10.30 [8.90, 11.95] | 10.37 [9.00, 11.79] | 0.996 | 0.004 | 10.44 [9.03, 12.20] | 10.43 [9.00, 11.85] | 0.433 | 0.05 |
| Platelets (×10^9/L) | 206.50 [149.50, 281.25] | 191.00 [134.28, 258.54] | 0.007 | 0.179 | 202.00 [146.58, 267.75] | 197.25 [137.62, 271.62] | 0.454 | 0.029 |
| APTT | 31.80 [27.50, 39.25] | 33.50 [28.59, 43.74] | 0.001 | 0.161 | 32.40 [27.80, 41.04] | 33.36 [28.40, 42.82] | 0.158 | 0.026 |
| BUN (mg/dL) | 25.67 [17.80, 44.00] | 25.20 [17.41, 40.90] | 0.146 | 0.142 | 25.09 [17.27, 40.00] | 26.62 [18.00, 44.19] | 0.524 | 0.027 |
| Creatinine (mg/dL) | 1.10 [0.70, 1.87] | 1.17 [0.80, 1.83] | 0.063 | 0.064 | 1.10 [0.73, 1.90] | 1.15 [0.80, 1.83] | 0.349 | 0.037 |
| Calcium (mg/dL) | 8.35 [7.85, 8.80] | 8.17 [7.68, 8.66] | <0.001 | 0.238 | 8.27 [7.77, 8.70] | 8.24 [7.75, 8.76] | 0.807 | 0.006 |
| Chloride (mmol/L) | 102.00 [97.50, 106.00] | 103.60 [99.50, 107.00] | 0.001 | 0.158 | 102.33 [98.35, 106.67] | 103.00 [99.00, 106.32] | 0.679 | 0.011 |
| Sodium (mmol/L) | 139.67 [136.33, 142.50] | 139.00 [136.00, 141.50] | 0.005 | 0.18 | 139.33 [135.67, 142.24] | 139.00 [136.43, 142.00] | 0.674 | 0.039 |
| Potassium (mmol/L) | 4.23 [3.87, 4.77] | 4.34 [3.90, 4.78] | 0.164 | 0.077 | 4.25 [3.90, 4.80] | 4.35 [3.90, 4.70] | 0.841 | 0.007 |
| **Medications and interventions** | | | | | | | | |
| Vasoactive agent (%) | 353 (68.81) | 451 (83.67) | <0.001 | 0.355 | 300 (76.92) | 307 (78.72) | 0.546 | 0.043 |
| Continuous renal replacement therapy (%) | 51 ( 9.94) | 89 (16.51) | 0.002 | 0.195 | 46 (11.79) | 52 (13.33) | 0.517 | 0.046 |
| Invasive arterial pressure monitoring (%) | 322 (62.77) | 428 (79.41) | <0.001 | 0.373 | 276 (70.77) | 287 (73.59) | 0.380 | 0.063 |
| Peripherally inserted central catheter (%) | 249 (48.54) | 245 (45.45) | 0.317 | 0.062 | 175 (44.87) | 184 (47.18) | 0.518 | 0.046 |
| **Disease severity scoring system** | | | | | | | | |
| SOFA | 7.00 [5.00, 10.00] | 7.00 [5.00, 10.00] | 0.434 | 0.036 | 7.00 [5.00, 10.00] | 7.00 [5.00, 10.00] | 0.882 | 0.02 |
| GCS | 15.00 [13.00, 15.00] | 15.00 [14.00, 15.00] | 0.834 | 0.052 | 15.00 [13.00, 15.00] | 15.00 [14.00, 15.00] | 0.822 | 0.034 |
| APS III | 54.00 [42.00, 68.00] | 55.00 [45.00, 71.00] | 0.038 | 0.129 | 56.00 [43.00, 69.00] | 54.50 [44.00, 67.75] | 0.857 | 0.001 |
| CCI | 6.00 [5.00, 8.00] | 6.00 [5.00, 8.00] | 0.018 | 0.132 | 6.00 [5.00, 8.00] | 6.00 [5.00, 8.00] | 0.359 | 0.034 |
| **Comorbidities** | | | | | | | | |
| Congestive heart failure (%) | 236 (46.00) | 271 (50.28) | 0.166 | 0.086 | 185 (47.44) | 190 (48.72) | 0.720 | 0.026 |
| Cerebrovascular_disease (%) | 82 (15.98) | 78 (14.47) | 0.495 | 0.042 | 62 (15.90) | 62 (15.90) | 1.000 | <0.001 |
| Liver disease (%) | 65 (12.67) | 90 (16.70) | 0.065 | 0.114 | 49 (12.56) | 59 (15.13) | 0.300 | 0.074 |
| Diabetes (%) | 180 (35.09) | 191 (35.44) | 0.906 | 0.007 | 141 (36.15) | 138 (35.38) | 0.823 | 0.016 |
| Renal disease (%) | 136 (26.51) | 136 (25.23) | 0.636 | 0.029 | 100 (25.64) | 103 (26.41) | 0.807 | 0.018 |
| Cancer (%) | 61 (11.89) | 79 (14.66) | 0.187 | 0.082 | 54 (13.85) | 50 (12.82) | 0.674 | 0.03 |


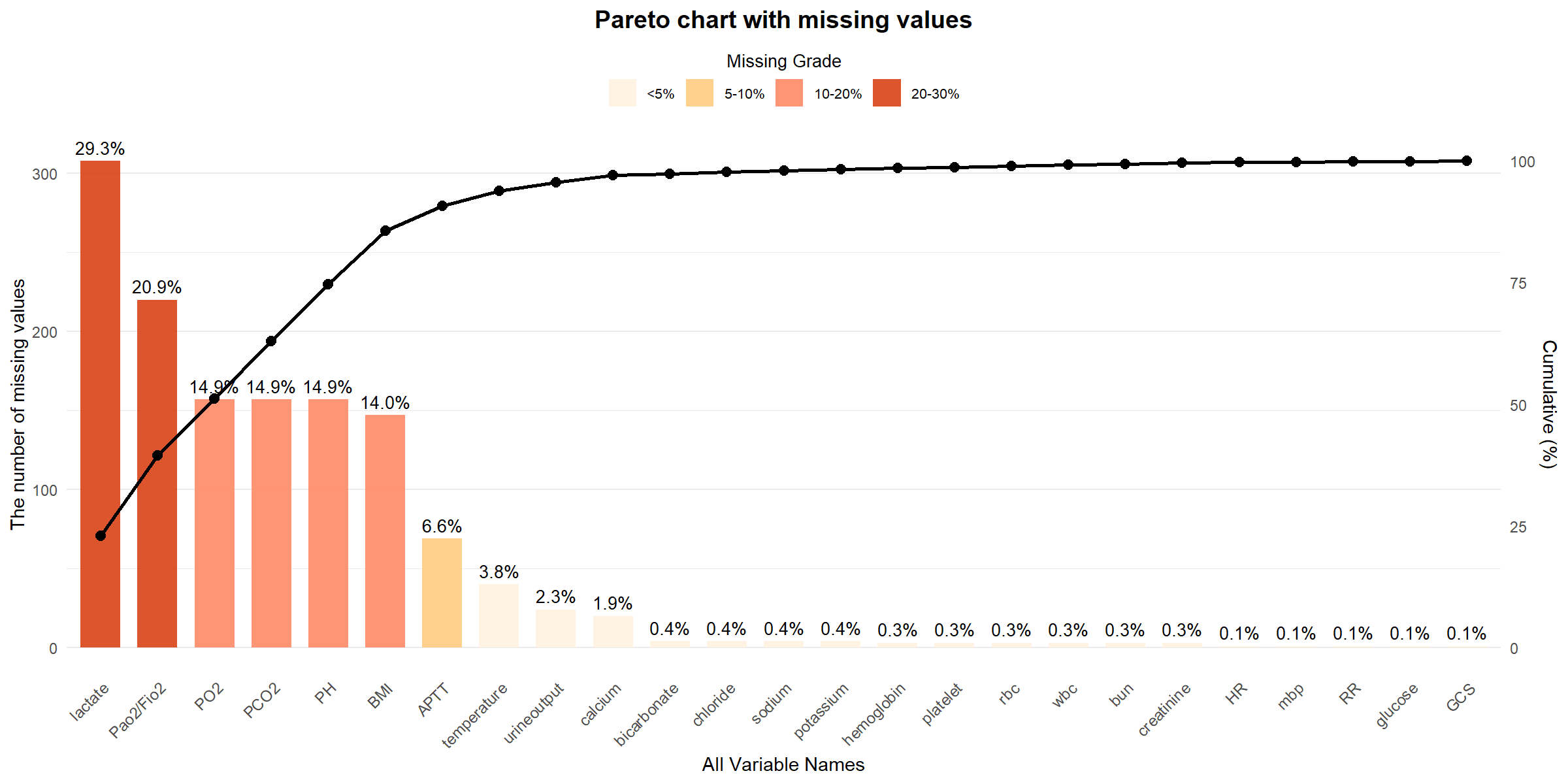


**Supplementary Figure S1**
